# Comparison Between the 24-hour Holter Test and 72-hour Single-Lead Electrocardiogram Monitoring With an Adhesive Patch-Type Device for Atrial Fibrillation Detection: Prospective Cohort Study

**DOI:** 10.2196/37970

**Published:** 2022-05-09

**Authors:** Soonil Kwon, So-Ryoung Lee, Eue-Keun Choi, Hyo-Jeong Ahn, Hee-Seok Song, Young-Shin Lee, Seil Oh, Gregory Y H Lip

**Affiliations:** 1 Department of Internal Medicine Seoul National University Hospital Seoul Republic of Korea; 2 Department of Internal Medicine Seoul National University College of Medicine Seoul Republic of Korea; 3 Seers Technology Co Seongnam-si Republic of Korea; 4 Liverpool Centre for Cardiovascular Science University of Liverpool Liverpool United Kingdom; 5 Department of Clinical Medicine Aalborg University Aalborg Denmark

**Keywords:** atrial fibrillation, diagnosis, electrocardiogram, wearable device, health monitoring, Holter, cardiac, arrhythmia, electrocardiogram, ECG, EKG, digital tool, cardiology, patient monitoring, outpatient clinic, cardiac health, diagnostic, patient, clinician, digital health

## Abstract

**Background:**

There is insufficient evidence for the use of single-lead electrocardiogram (ECG) monitoring with an adhesive patch-type device (APD) over an extended period compared to that of the 24-hour Holter test for atrial fibrillation (AF) detection.

**Objective:**

In this paper, we aimed to compare AF detection by the 24-hour Holter test and 72-hour single-lead ECG monitoring using an APD among patients with AF.

**Methods:**

This was a prospective, single-center cohort study. A total of 210 patients with AF with clinical indications for the Holter test at cardiology outpatient clinics were enrolled in the study. The study participants were equipped with both the Holter device and APD for the first 24 hours. Subsequently, only the APD continued ECG monitoring for an additional 48 hours. AF detection during the first 24 hours was compared between the two devices. The diagnostic benefits of extended monitoring using the APD were evaluated.

**Results:**

A total of 200 patients (mean age 60 years; n=141, 70.5% male; and n=59, 29.5% female) completed 72-hour ECG monitoring with the APD. During the first 24 hours, both monitoring methods detected AF in the same 40/200 (20%) patients (including 20 patients each with paroxysmal and persistent AF). Compared to the 24-hour Holter test, the APD increased the AF detection rate by 1.5-fold (58/200; 29%) and 1.6-fold (64/200; 32%) with 48- and 72-hour monitoring, respectively. With the APD, the number of newly discovered patients with paroxysmal AF was 20/44 (45.5%), 18/44 (40.9%), and 6/44 (13.6%) at 24-, 48-, and 72-hour monitoring, respectively. Compared with 24-hour Holter monitoring, 72-hour monitoring with the APD increased the detection rate of paroxysmal AF by 2.2-fold (44/20).

**Conclusions:**

Compared to the 24-hour Holter test, AF detection could be improved with 72-hour single-lead ECG monitoring with the APD.

## Introduction

Electrocardiogram (ECG) monitoring is essential for the detection of atrial fibrillation (AF). Although a standard 12-lead ECG can be used to detect AF, its diagnostic effectiveness decreases as the AF burden becomes low and multiple snapshots of 12-lead ECGs or ambulatory ECG monitoring are often required [1,2]. Although numerous handheld or wearable ECG devices are now readily available [3], the Holter test remains the gold standard for ambulatory ECG monitoring. Briefly, the Holter test is usually performed over 24 hours and can record multiple ECG leads. However, in the case of paroxysmal AF, the known AF burden is generally less than 5% [4]. In such cases, more extended ECG monitoring is usually necessary to detect AF.

Recently, adhesive patch-type devices (APDs) have been used to detect AF. Compared to the Holter test, APDs are generally more compact and convenient for patients [5]. APDs also have the advantage of an extended monitoring period for up to several days, depending on the product. Therefore, APDs could be a valuable alternative to the Holter test. However, most APDs monitor single-lead ECG such that they can record ECG signals along a single vector. As a result, there are concerns of over- or under-detection of AF compared to the standard Holter test [6]. Additionally, the diagnostic performance of single-lead ECG monitoring could be suboptimal due to noisy tracings, frequent ectopic beats, or the coexistence of other tachyarrhythmias [7]. Although multiple studies have validated the diagnostic performance of single-lead ECG monitoring with APDs for various cardiac arrhythmias [6,8-11], evidence of direct comparisons between the Holter test and single-lead ECG monitoring with an APD for AF detection remains limited [12].

This study aimed to compare the 24-hour Holter test to 72-hour single-lead ECG monitoring with an APD among patients with AF in routine medical care.

## Methods

### Ethics Approval

The study protocol was approved by the Seoul National University Hospital Institutional Review Board and adhered to the Declaration of Helsinki revised in 2013 (IRB No: H-2006-224-1138).

### Study Design and Population

This was a single-center, prospective cohort study. Among the patients who received outpatient management for AF at our institution (Seoul National University Hospital, Seoul, Republic of Korea), the patients who needed ambulatory ECG monitoring for AF management or evaluation were screened for the study. All patients were medically examined and screened by any of the 3 electrophysiologists (EKC, SRL, or SO). Screening and recruitment processes were conducted in the outpatient clinic setting.

The *inclusion criteria* of the study population were (1) those who were previously diagnosed with AF and (2) those who were indicated for the 24-hour Holter test for routine management or monitoring of AF at outpatient clinics. The *exclusion criteria* were (1) persistent atrial flutter or atrial tachycardia and (2) failure to complete simultaneous single-lead ECG monitoring with the APD and Holter test for the first 24 hours.

Between October 2020 and September 2021, a total of 210 patients were enrolled in the study. Among them, 2 (1%) patients had no AF but persistent atrial tachycardia, and 8 (3.8%) patients did not complete simultaneous monitoring for the first 24 hours due to detachment of the monitoring device or recording errors. Therefore, a total of 200 participants were included in this study.

### Study Flow

After obtaining informed consent, baseline characteristics were examined by a researcher in the outpatient clinic. Baseline characteristics included demographic information (age, sex, height, body weight, and body mass index), information on AF (types of AF, CHA_2_DS_2_-VASc [congestive heart failure, hypertension, age ≥75 years, diabetes mellitus, stroke or transient ischemic attack, vascular disease, age 65 to 74 years, sex category] scores, and history of treatment for AF), comorbidities (hypertension, diabetes mellitus, heart failure, vascular disease, chronic kidney disease, liver disease, and thromboembolism), and concomitant medications (antiarrhythmic agents, diuretics, oral anticoagulants, and antiplatelet agents).

**Figure 1:**
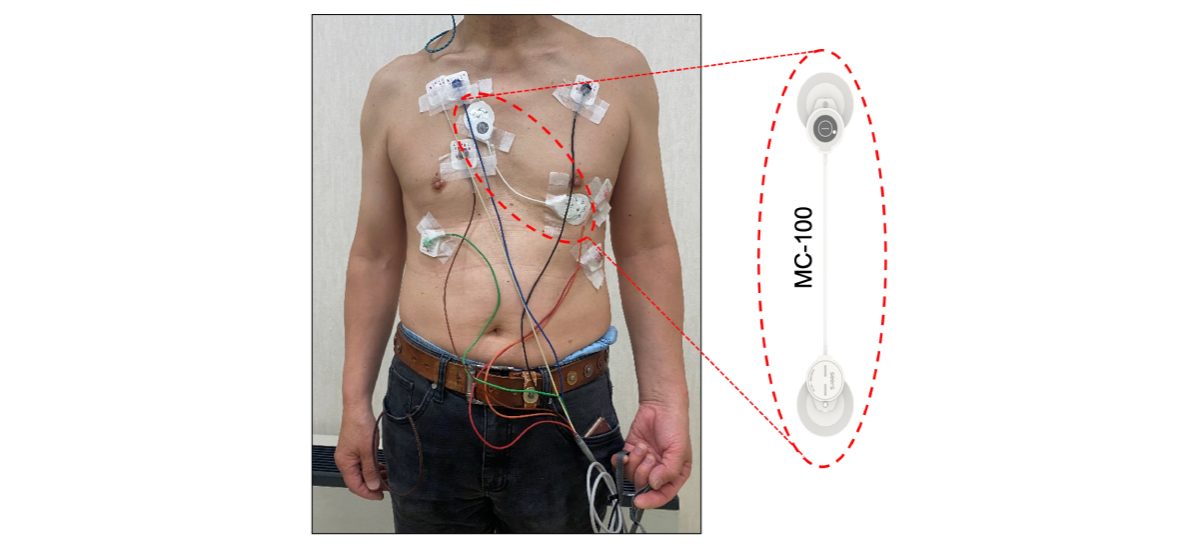
Device setting for electrocardiogram monitoring of a study participant. A study participant recorded a single-lead electrocardiogram (lead II) using an adhesive patch-type device (MC-100) and a three-channel electrocardiogram (lead I, V1, and V6) using the Holter test. Overlap of the electrodes of both devices was avoided to prevent signal noise and interference.

After enrollment, each participant started simultaneous single-lead ECG monitoring and Holter tests for the first 24 hours. An APD (mobiCARE MC-100, Seers technology, Seongnam-si, Gyeonggi-do, Republic of Korea) and a Holter device (SEER Light, GE Healthcare, Chicago, IL, USA) were attached to each participant, as shown in Figure 1. The Holter device was set to record three channels (leads I, V1, and V6), and the electrodes were placed at the positions for standard ECG measurement. The APD was set to record a single channel (lead II) and was placed 45 from the internipple line. Overlap of the electrodes in both devices was avoided to prevent signal noise and interference.

After completing the simultaneous monitoring for 24 hours, participants returned the Holter device and continued single-lead ECG monitoring with the APD for additional 48 hours. After completing the comprehensive monitoring, each participant returned the APD and responded to a survey on the convenience of using the APD. The raw data of both devices were extracted and anonymized to protect participants’ privacy. Raw data were independently reviewed and analyzed by 4 cardiologists (SK, SRL, EKC, and HJA). If there was any discrepancy in the interpretation of the ECG signal, the senior electrophysiologist (EKC) decided the final interpretation.

### A Brief Specification of MC-100

The APD used in the study (MC-100) has two medical standard 4.0 mm electrode snaps connected by a single wire. The device is compatible with conventional sticky ECG electrodes. It is powered by a commercial CR2032H coin cell battery and can operate continuously for at least 72 hours. The size of the device is 29 mm × 120 mm, and it weighs 8.9 grams. The device can record a single-lead ECG signal with a sampling rate of 256 Hz. Additionally, the device has accelerometers and gyroscopes to measure movement activity. The device is connected to the user’s smartphone using Bluetooth and transmits ECG data to the smartphone. A user can access ECG data from a smartphone, and real time monitoring is possible using a preinstalled app. The MC-100 has a built-in memory of 256 kilobytes, which can store ECG data for up to 2-3 minutes if it is disconnected from the smartphone. During validation of the ECG measurements using the MC-100 for the population with non-AF cardiac arrhythmias, the device showed a diagnostic performance comparable to that of a conventional Holter test [13].

### Sample Size Determination

We used the McNemar test to estimate the sample size. Based on previous reports [5,8], we assumed that 14% of patients would be negative for the Holter test but positive for the APD due to the extended monitoring period, while 4% of patients would be positive for the Holter test but negative for the APD due to potential disadvantages of single-lead ECG monitoring. To achieve a power of 80% and a two-sided significance of 5%, the study required 194 participants. Considering potential dropouts, we estimated a total of 200 participants are required to conduct the study. The PASS 15 Power Analysis and Sample Size Software was used to perform sample size calculations.

### Statistical Analysis

The diagnostic performances of the APD and Holter tests were compared. The variables for the comparison included total monitoring time (minutes), the proportion of noise (ie, uninterpretable portions of the recorded signals, %), AF detection rate (%), and AF burden (%). To compare the variables between the two tests, a paired *t* test or Wilcoxon signed-rank test was performed according to their normality. The AF detection rate and AF burden were measured every 24 hours to observe the diagnostic benefits of extended single-lead ECG monitoring daily. We also recalculated the AF detection rate and AF burden for the APD by only including AF episodes that lasted ≥30 seconds. In all statistical analyses, a *P* value of less than .05 was considered statistically significant. Statistical analyses were performed using SPSS Statistics for Windows, version 22.0 (IBM Corp).

## Results

### Baseline Characteristics

The baseline characteristics of the study population are presented in Table 1. The mean patient age was 60 years, and 70.5% (141/200) of the patients were male. The proportions of paroxysmal AF and persistent AF were 68% (136/200) and 32% (64/200), respectively. The most common comorbidity was hypertension (54.5%, 109/200). Most participants used beta-blockers (33.5%, 67/200), oral anticoagulants (57%, 114/200), and class Ic antiarrhythmic agents (43%, 86/200).

**Table 1:** Baseline characteristics of the study population (N=200).

Characteristics		Value
Age (year), mean (SD)		60 (7.8)
**Gender, n (%)**		
	Male	141 (70.5)
	Female	59 (29.5)
Intersex, n (%)		0 (0)
Height, cm (SD)		167.2 (7.9)
Weight, kg (SD)		70.2 (11)
Body mass index, kg/m^2^ (SD)		25.1 (3.1)
Mean CHA_2_DS_2_-VASc^a^ score (SD)		1.5 (1.1)
Median CHA_2_DS_2_-VASc score (IQR)		1 (1-2)
**AF^b^ types, n (%)**		
	Paroxysmal	136 (68)
	Persistent	64 (32)
**AF treatment information, n (%)**		
	Prior electrical cardioversion	53 (26.5)
	Prior catheter ablation	122 (61)
**Comorbidities, n (%)**		
	Hypertension	109 (54.5)
	Diabetes mellitus	34 (17)
	Heart failure	18 (9)
	Peripheral artery disease	1 (0.5)
	Chronic kidney disease	2 (1)
	Chronic liver disease	3 (1.5)
	Thromboembolism	2 (1)
**Concomitant medications, n (%)**		
	Beta-blocker	67 (33.5)
	Calcium channel blocker	39 (19.5)
	RAAS^c^ blockade	50 (25)
	Diuretics	11 (5.5)
	Oral anticoagulant	114 (57)
	Antiplatelet agent	25 (12.5)
	Class Ic antiarrhythmic agent	86 (43)
	Amiodarone	29 (14.5)

^a^CHA_2_DS_2_-VASc: congestive heart failure, hypertension, age ≥75 years, diabetes mellitus, stroke or transient ischemic attack, vascular disease, age 65 to 74 years, sex category.

^b^AF: atrial fibrillation.

^c^RAAS: renin-angiotensin-aldosterone system.

### Comparisons of ECG Monitoring Between the Holter and APD

A total of 200 participants performed ECG monitoring with the Holter and APD. The mean monitoring durations were 1402 (SD 106) min (0.97, SD 0.07 days) and 4242 (SD 401) min (2.95, SD 0.28 days) for the Holter and APD, respectively (*P*<.001; Table 2). The median noise proportions were significantly higher in the APD (median <0.1%, 95% CI 0-0.2 for the Holter; and median 0.3%, 95% CI 0.1-0.7 for the APD*,* *P*<.001). Most signal noises were caused by motion artifacts, touching of the device, or poor electrode contact. The APD had additional signal loss due to Bluetooth disconnection from the user’s smartphone (median 2%, 95% CI 1.0-4.4).

**Table 2:** Comparisons of ECG^a^ monitoring durations and noise proportions between the Holter and the adhesive patch-type device.

	24-hour Holter monitoring	72-hour single-lead ECG monitoring with an adhesive patch-type device	*P* value
Total participants, N	200	200	N/A^b^
Mean monitoring duration, min (SD)	1402 (106)	4242 (401)	<.001
Median noise proportions, % (IRQ)	<0.1 (0-0.2)	0.3 (0.1-0.7)	<.001

^a^ECG: electrocardiogram.

^b^N/A: not applicable.

### Feasibility of 72-Hour ECG Monitoring With the APD

Of the 200 participants, 188 (94%) completed the 72-hour ECG monitoring with the APD. During the extended monitoring period, 12 (6%) participants failed to complete the 72-hour ECG monitoring. Reasons for failing to complete the 72-hour monitoring included device or app errors in 4 (2%) participants, misuse of the device by the user in 3 (1.5%) participants, skin irritation in 2 (1%) participants, and other reasons in 3 (1.5%) participants. The skin irritation that occurred in the 2 participants recovered spontaneously after removing the APD and did not require further medical aid.

### Comparisons of AF Detection and AF Burdens Between the Holter and APD

**Figure 2:**
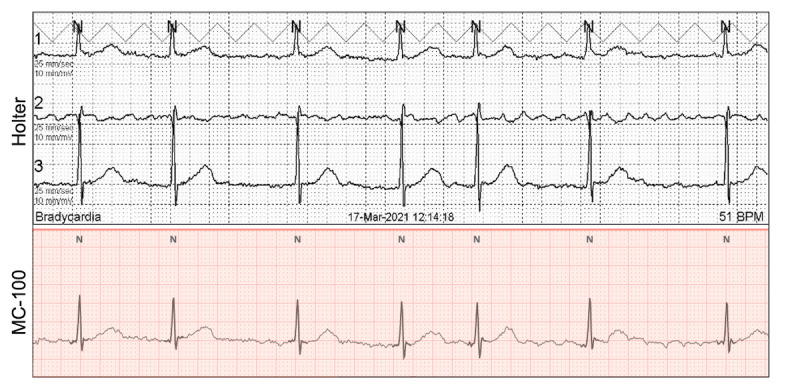
An example of persistent AF (participant #105) detected by the Holter and adhesive patch-type device. Both the Holter and adhesive patch-type device detected AF coherently. AF: atrial fibrillation.

**Figure 3:**
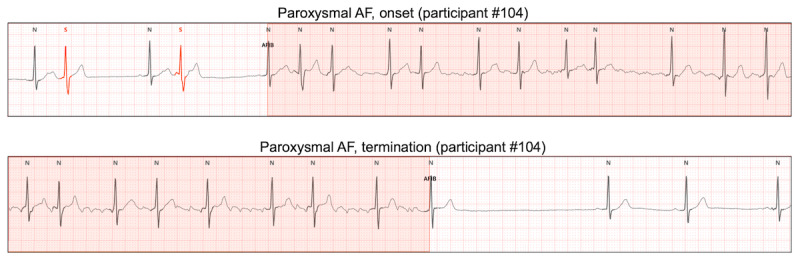
An example of onset and termination of paroxysmal AF detected by the adhesive patch-type device. Both onset and termination of paroxysmal AF can be accurately detected by the adhesive patch-type device. AF: atrial fibrillation.

**Figure 4:**
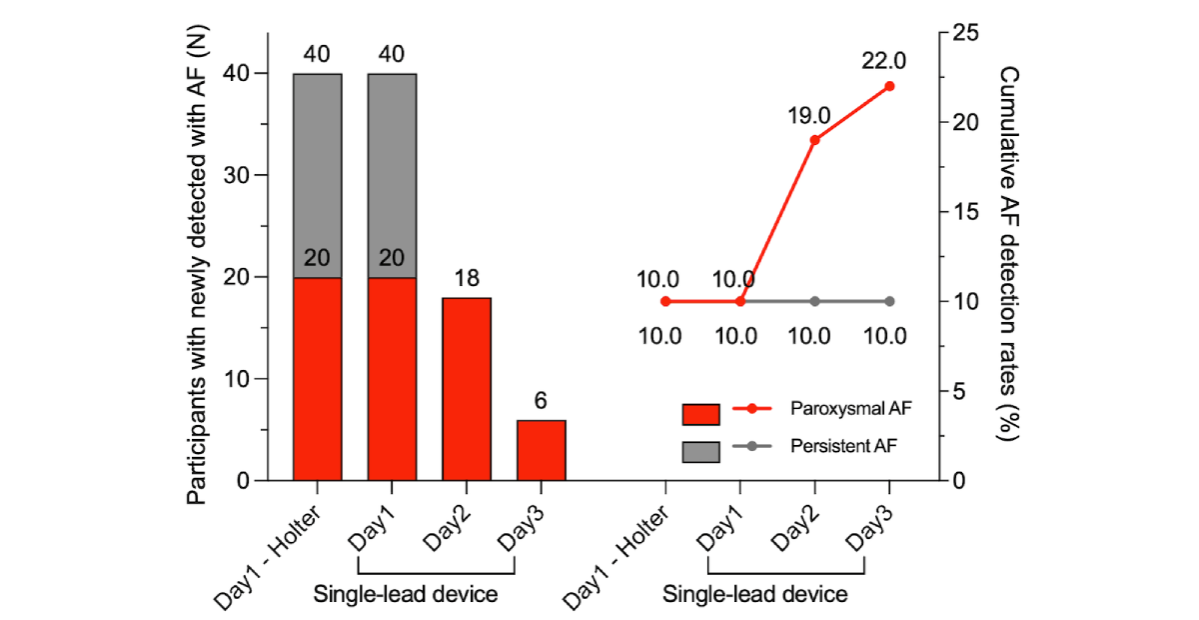
Comparison of AF detection between the Holter and adhesive patch-type device. The daily proportions of participants with AF were detected by the 24-hour Holter test and 72-hour single-lead electrocardiogram monitoring with the adhesive patch-type device. AF: atrial fibrillation.

Examples of single-lead ECG monitoring with the APD for persistent and paroxysmal AF are presented in Figure 2 and Figure 3, respectively. For the first 24 hours, both the Holter and APD yielded the same AF detection rate (40/200, 20% of participants; Figure 4). Paroxysmal and persistent AF were equally identified in 20 participants using both devices. During the extended monitoring period, the APD detected paroxysmal AF in 18 (9%) and 6 (3%) new participants on days 2 and 3, respectively. Compared to the 24-hour Holter test, 72-hour ECG monitoring with the APD increased the AF detection rate by 1.6-fold (40/200, 20% with the Holter; and 64/200, 32% with the APD). When comparing only participants with paroxysmal AF, the APD increased the AF detection rate by 2.2-fold (20/180, 11.1% with the Holter; and 44/180, 24.4% with the APD).

**Figure 5:**
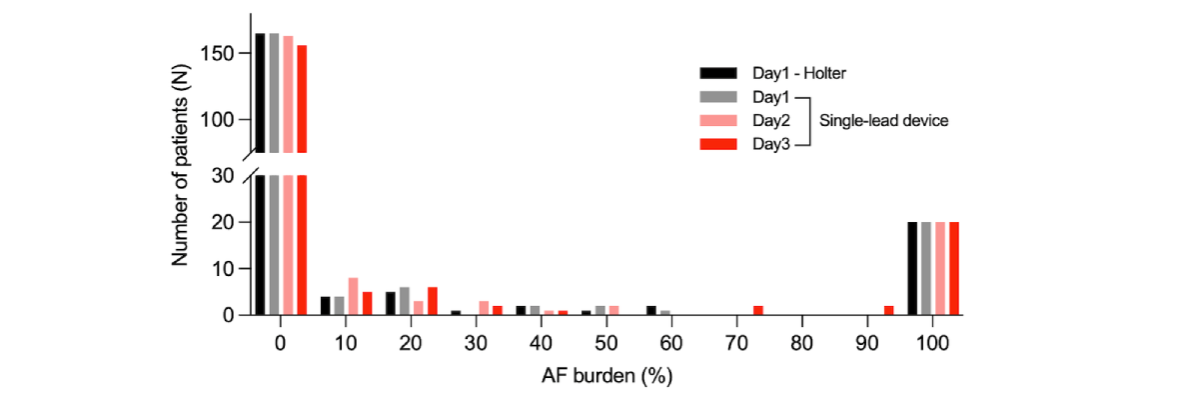
Distribution of AF burden measured by the Holter and adhesive patch-type device. The daily AF burdens were compared between 24-hour Holter monitoring and 72-hour single-lead electrocardiogram monitoring with the adhesive patch-type device. AF: atrial fibrillation.

**Figure 6:**
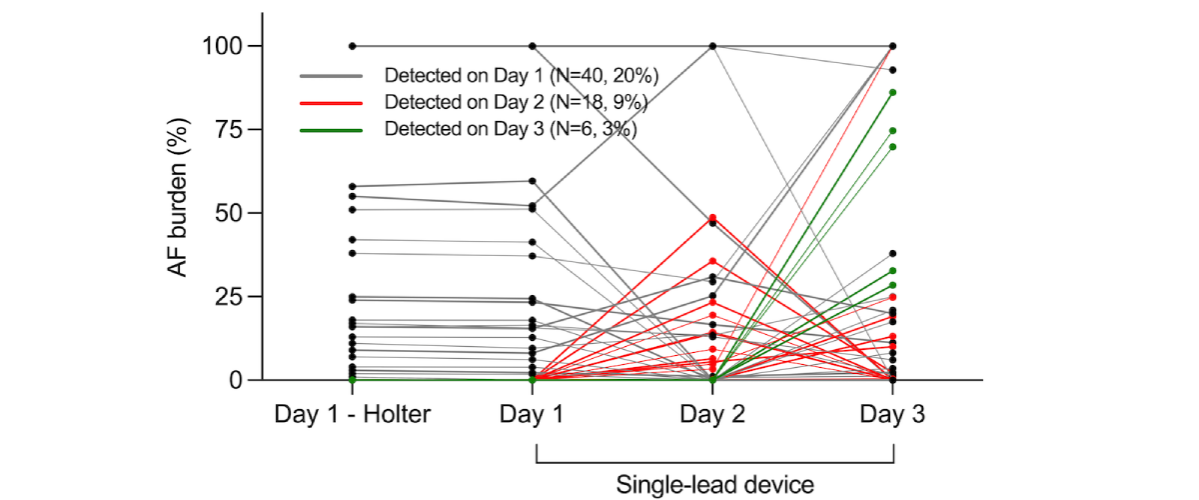
Dynamic changes of daily AF burden. For each participant, daily AF burden was tracked over the monitoring period using the adhesive patch-type device. AF: atrial fibrillation.

The daily distributions of AF burden measured by the 24-hour Holter test and 72-hour single-lead ECG monitoring with the APD are compared in Figure 5. There was no significant difference in the AF burden measured by the two devices on day 1 (*P*=.06). Except for the participants with persistent AF (ie, AF burden of 100%), most AF burdens were less than 5% (165/180, 91.7% on day 1; 163/180, 90.6% on day 2; 156/180, 86.7% on day 3 with the APD). The next most common AF burden was 5%-25% (10/180, 5.5% on day 1; 11/180, 6.1% on days 2 and 3 with the APD). Individual AF burdens changed dynamically over the monitoring period, except in cases of persistent AF (Figure 6).

### Impact of the Duration of AF Episodes on AF Detection

**Figure 7:**
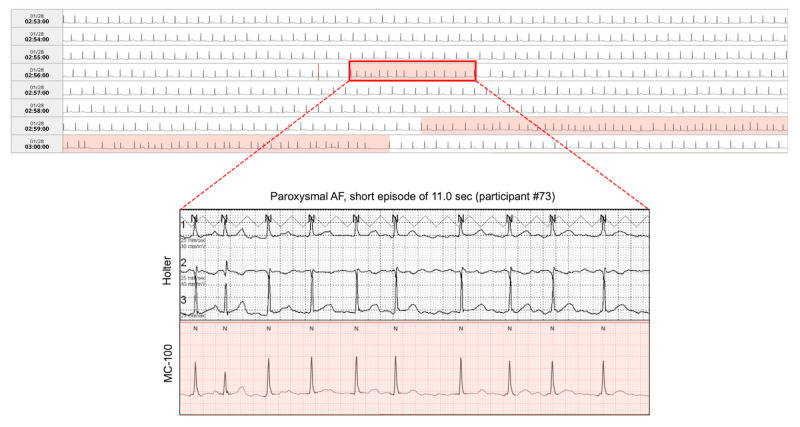
An example of short episode of paroxysmal AF detected by the Holter and adhesive patch-type device. Both the Holter and adhesive patch-type device detected a short episode of paroxysmal AF accurately. AF: atrial fibrillation.

**Figure 8:**
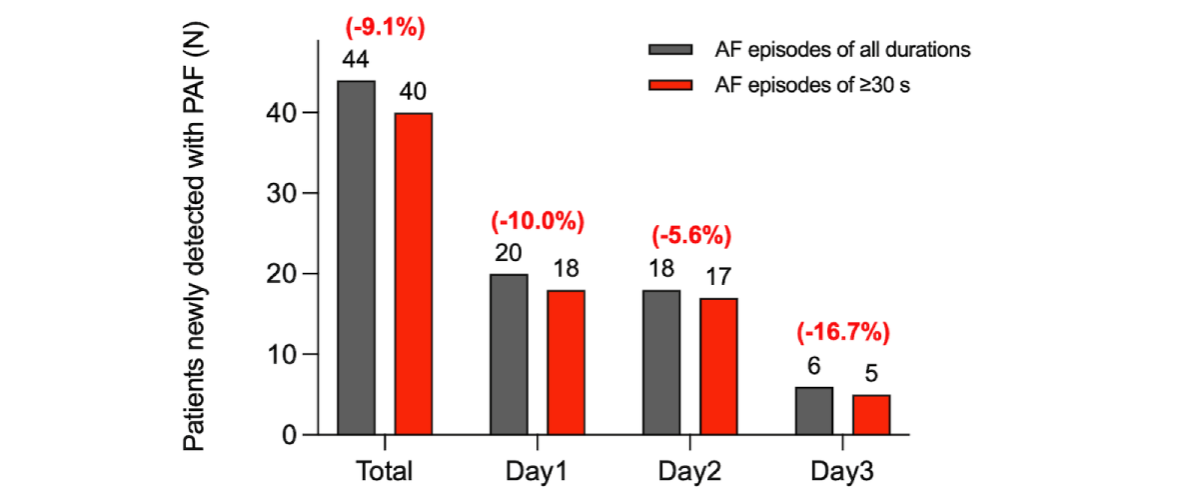
Comparison of the detection rates of paroxysmal AF with the adhesive patch-type device (MC-100) by episode durations. Limiting the minimally required duration of AF episodes to 30 seconds decreased the detection rate of paroxysmal AF by 9.1% overall. AF: atrial fibrillation; PAF: paroxysmal atrial fibrillation.

An example of a short episode of paroxysmal AF (duration <30 seconds) is shown in Figure 7. If only episodes lasting over 30 seconds with the APD were counted as AF, the detection rate of paroxysmal AF is presented in Figure 8. Limiting the minimally required duration of AF episodes to 30 seconds decreased the detection rate of paroxysmal AF by 9.1% overall. Despite the decrease in the detection rate of paroxysmal AF, 72-hour single-lead ECG monitoring with the APD yielded a 2-fold higher detection rate than the 24-hour Holter test (20/180, 11.1% with the Holter; and 40/180, 22.2% with the APD).

### The Convenience of Using the APD for Extended ECG Monitoring

The survey results for the use of the APD are presented in Multimedia Appendix 1. The surveys were collected by 190 study participants (190/200, 95%). Most participants did not report discomfort or skin irritability during use of the APD (66/190, 34.7% and 65/190, 34.2%, respectively). Among respondents, 27/190 (14.2%) reported skin irritability. Instances of discomfort using the APD were the most frequent during activity (54/190, 28.4%), followed by that during sleep (35/190, 18.4%). Episodes of intermittent device detachment were observed in 111/190 (58.4%) of the respondents. Overall, more than a half of the respondents responded very positively to using the device and app (107/190, 56.3% and 98/190, 51.6%, respectively).

## Discussion

### Principal Findings

This study compared AF detection rates with a 24-hour Holter test and 72-hour single-lead ECG monitoring with an APD among patients requiring AF monitoring. The principal findings of the study are as follows: (1) during the simultaneous use of both monitoring methods, they yielded the same AF detection rate over 24 hours; (2) extended monitoring with the APD increased AF detection rates by 1.6-fold compared to those with the 24-hour Holter test (2.2-fold for the case of paroxysmal AF); (3) limiting the minimally required duration of AF episodes to 30 seconds decreased the detection rate of paroxysmal AF by 9.1% with the APD; and (4) most participants responded that it was convenient to use the APD over the extended monitoring period.

The major differences between our study and others are that (1) we compared the AF detection rate between the Holter test and the APD from routine medical care for patients with AF, and (2) we evaluated the impact of the duration of AF episodes on AF detection using the APD.

When managing patients with AF in an outpatient setting, performing a 24-hour Holter test is common. However, we found that the 24-hour Holter test detected only approximately 40/64 (62.5%) of participants with AF compared to those by the 72-hour single-lead ECG monitoring. This result suggests that the outpatient-based 24-hour Holter test is often ineffective for AF detection. Therefore, the management or evaluation of patients with AF might be suboptimal when the 24-hour Holter test is used, especially in cases of paroxysmal AF.

According to recent European guidelines [1], AF-like episodes of at least 30 seconds are required to diagnose AF using a single-lead ECG device. It is challenging to identify P waves as accurately as a standard 12-lead ECG using a single-lead ECG. This study found that identifying AF episodes lasting ≥30 seconds decreased the AF detection rate by 9.1%. Nevertheless, 72-hour single-lead ECG monitoring was superior to that of the 24-hour Holter test for AF detection.

ECG monitoring is an essential method for AF detection. Recently, wearable or portable ECG monitoring devices have become widely accepted for AF detection [14]. Smartwatches with the capability of ECG measurement and APDs are typical examples of newly introduced ECG monitoring tools [3]. Unlike smartwatches or handheld devices, APDs can continuously monitor ECG signals; therefore, APDs have a potential to maximize AF detection rates during a given monitoring period. Some APDs can record multiple-lead ECG, but most devices have been designed to record single-lead ECG to minimize their size and maximize their convenience. The convenience of APDs is that they can monitor ECG signals for an extended period (several days to weeks), and thus increase the possibility of AF detection without disturbing the patient. In addition, they are small and convenient to use [5]; however, one disadvantage is that most APDs can only record single-lead ECG signals. Consequently, if a patient has P waves that are low in amplitude along with the vector between the device’s electrodes, there is a possibility of misdiagnosing atrial arrhythmias, including AF [6,13]. However, in this study, both the Holter test and the APD showed equivalent AF detection rates during the first 24 hours. One possible explanation is that the number of patients might have been insufficient to show the difference in the AF detection rates between the two monitoring methods during the first 24 hours. However, the APD might have been as effective as Holter because the APD detected P waves effectively in our study; the APD’s electrodes were attached 120 mm apart and along the P wave axis to increase the detection of P waves. A further study is warranted regarding the effectiveness of P wave detection with the APD.

Some studies investigated the use of APDs for AF detection [6,15,16]. A systematic review by Ramkumar et al [17] found that a moderate linear relationship exists between monitoring time and an AF detection rate for a single-lead ECG device. Although a more extended monitoring with APDs would increase the AF detection rate, the mSToPS trial found that most AF detection occurred within a week [15]. However, as the monitoring period becomes longer, test compliance would decrease while the possibility of skin problems caused by APDs would increase. For example, Heckbert et al [9] reported that APDs with a median monitoring time of 14 days induced skin irritation in 4% of the participants. Similar to the mSToPS trial, this study also found that most AF detection occurred within 7 days. In our study, only 1% (N=2) of the study participants discontinued ECG monitoring with the APD (MC-100) because of skin problems. Skin irritation occurred less commonly in this study than in other studies due to differences in the monitoring period with the APDs [9,10]. In addition, the MC-100 uses conventional ECG snap electrodes that are widely used for ECG measurements, and the contact area between the device and the skin is smaller than that of other commercial products. The smaller contact area of adhesives might also have contributed to a lower prevalence of skin problems in our study. Moreover, the feasibility of extended monitoring with an APD could be an issue due to device detachment during daily activity. However, the APD used in our study was easy to reattach to the body because the device was small and had a simple and lightweight structure. Therefore, in most cases, the detachment period was relatively short. As a result, the proportion of signal noise due to any detachment episodes accounted for only a median of 0.3% (95% CI 0.1-0.7) of the total monitoring time.

### Limitations

This study has several limitations. First, it focused on the population diagnosed with AF who received routine medical care at outpatient clinics. Therefore, the diagnostic performance in the general population cannot be estimated. Second, during the extended monitoring period, there were possible false-positive or false-negative episodes with the APD because no Holter data or standard 12-lead ECG was available to validate the episodes. False-positive AF episodes could also be attributed to underdetected ectopic P waves or premature atrial beats with the single-lead ECG data [6,13]. Third, this study cannot determine the AF detection performance of the APD for special cases including concomitant complete atrioventricular block or slow ventricular response as the study participants did not have such cases.

### Conclusions

Compared to the 24-hour Holter test, 72-hour single-lead ECG monitoring with an APD could improve AF detection rates. Both tests were equally effective during the first 24 hours despite the potential disadvantages of single-lead ECG monitoring. Focusing on paroxysmal AF, the detection rates could be improved by 2.2-fold with the APD. In addition, the APD was convenient for extended monitoring without causing serious skin irritation. Our results showed that the extended monitoring with the APD for AF detection was feasible and had good compliance. Extended monitoring of single-lead ECG with the APD could be beneficial for AF detection among patients whereby conventional ECG tests were inadequate in documenting AF episodes.

## Appendix

Multimedia Appendix 1 The survey on the use of MC-100 (English-translated version).

## Abbreviations

AF: atrial fibrillation
APD: adhesive patch-type device
ECG: electrocardiogram
