# Supplementary material for: Comparison Between the 24-hour Holter Test and 72-hour Single-Lead Electrocardiogram Monitoring With an Adhesive Patch-Type Device for Atrial Fibrillation Detection: Prospective Cohort Study
Source: J Med Internet Res. 2022 May 9;24(5):e37970. doi: 10.2196/37970 (PMC9127648; doi:10.2196/37970)
Supplement: Multimedia Appendix 1 [file jmir_v24i5e37970_app1.docx]

**Multimedia Appendix 1**

**Table S1. The survey on the use of MC-100 (English-translated version)**

| **Convenience of using MC-100** | No | Minimally | Sometimes | Much | | Very much |
| --- | --- | --- | --- | --- | --- | --- |
| Did you feel discomfort with the device? | 66 (34.7%) | 54 (28.4%) | 34 (17.9%) | 30 (15.8%) | | 6 (3.2%) |
| Did you feel skin irritability with the device? | 65 (34.2%) | 31 (16.3%) | 21 (11.1%) | 46 (24.2%) | | 27 (14.2%) |
| When do you feel the device discomfort? (multiple responses possible) | | | | | | |
| None | 76 (40.0%) | | | | | |
| During sleep | 35 (18.4%) | | | | | |
| During activity | 54 (28.4%) | | | | | |
| During rest | 32 (16.8%) | | | | | |
| Did you have device detachment(s) during the monitoring period? | 111 (58.4%) | | | | | |
| **Usability of the smartphone application for the monitoring device** | None | Few | Sometimes | Often | Very often | |
| How do you often check the application for monitoring your ECG? | 27 (11.6%) | 11 (5.8%) | 46 (24.2%) | 44 (23.2%) | 62 (32.6%) | |
| Did you record an episode at the application when you had symptom(s)? | 89 (46.8%) | 17 (8.9%) | 37 (19.5%) | 19 (10.0%) | 28 (14.7%) | |
| **Overall evaluation on the use of MC-100** | Very negative | Negative | Equivocal | Positive | | Very positive |
| Do you satisfy with using the device? | 2 (1.1%) | 10 (5.3%) | 30 (15.8%) | 41 (21.6%) | 107 (56.3%) | |
| Do you satisfy with using the application? | 3 (1.6%) | 9 (4.7%) | 33 (17.4%) | 47 (24.7%) | 98 (51.6%) | |
